# Predictors of transportation delay in patients with suspected ST-elevation-myocardial infarction in the VIENNA-STEMI network

**DOI:** 10.1007/s00392-019-01520-z

**Published:** 2019-06-29

**Authors:** Bernhard Jäger, Paul Michael Haller, Edita Piackova, Alfred Kaff, Günter Christ, Wolfgang Schreiber, Franz Weidinger, Thomas Stefenelli, Georg Delle-Karth, Gerhard Maurer, Kurt Huber, for the Vienna STEMI Registry Group

**Affiliations:** 1 3rd Medical Department, Cardiology and Intensive Care Medicine, Wilhelminen Hospital, Montleartstrasse 35-37, 1160 Vienna, Austria; 2 Medical Faculty, Sigmund Freud Private University, Vienna, Austria; 3 Medical University of Vienna, Vienna, Austria; 4 Ambulance Services Vienna, Vienna, Austria; 5 5th Medial Department, Cardiology, Sozialmedizinsiches Zentrum Süd - Kaiser-Franz-Josef-Spital, Vienna, Austria; 6 Department of Emergency Medicine, Medical University of Vienna, Vienna, Austria; 7 2nd Medical Department, Cardiology, Krankenhaus Rudolfstiftung, Vienna, Austria; 8 1st Medical Department, Cardiology, Sozialmedizinisches Zentrum Ost, Vienna, Austria; 9 4th Medical Department, Cardiology, Krankenhaus Hietzing, Vienna, Austria; 10 Department of Cardiology, Medical University of Vienna, Vienna, Austria

**Keywords:** ST-elevation myocardial infarction, STEMI, Emergency medical system, EMS, System delay, Transportation delay

## Abstract

**Objective:**

The emergency medical service (EMS) provides rapid pre-hospital diagnosis and transportation in ST-elevation myocardial infarction (STEMI) systems of care. Aim of the study was to assess temporal and regional characteristics of EMS-related delays in a metropolitan STEMI network.

**Methods:**

Patient call-to-arrival of EMS at site (call-to-site), transportation time from site to hospital (site-to-door), call-to-door, patient’s location, month, weekday, and hour of EMS activation were recorded in 4751 patients referred to a tertiary center with suspicion of STEMI.

**Results:**

Median call-to-site, site-to-door, and call-to-door times were 9 (7–12), 39 (31–48), and 49 (41–59) minutes, respectively. The shortest transportation times were noted between 08:00 and 16:00 and in general on Sundays. They were significantly prolonged between midnight and 04:00, whereby the longest difference did not exceed 4 min in median. Patient’s site of call had a major impact on transportation times, which were shorter in Central and Western districts as compared to Southern and Eastern districts of Vienna (*p* < 0.001 between-group difference for call-to-site, site-to-door, and call-to-door). After multivariable adjustment, patient’s site of call was an independent predictor of call-to-site delay (*p* < 0.001). Moreover, age and hour of EMS activation were the strongest predictors of call-to-site, site-to-door, and call-to-door delays (*p* < 0.05).

**Conclusion:**

In our Viennese STEMI network, the strongest determinants of pre-hospital EMS-related transportation delays were patient’s site of call, patient’s age, and hour of EMS activation. Due to the significant but small median time delays, which are within the guideline-recommended time intervals, no impact on clinical outcome can be expected.

**Graphic abstract:**

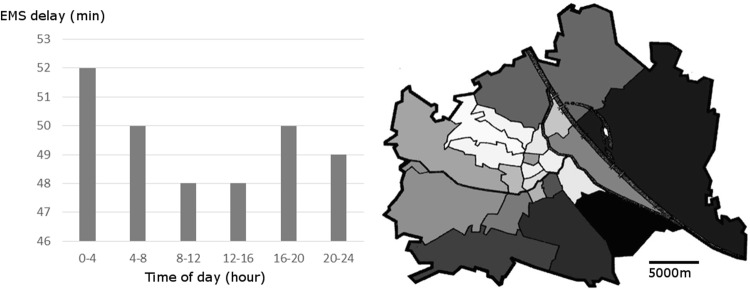

## Introduction

Timely reperfusion therapy is the cornerstone of successful treatment for ST-elevation myocardial infarction (STEMI) and is known to improve patient’s outcome [1–3]. Specific STEMI networks, consisting of an emergency ambulance medical system (EMS) staffed with emergency physicians or paramedics and tertiary hospitals with the capability of primary percutaneous coronary intervention (PCI), have been developed and established to shorten system-related delay in urban as well as in rural areas [4, 5]. Theoretically, the greatest benefits can be achieved by shortening the pre-hospital delay, as it usually accounts for the largest proportion of total ischemic time in STEMI [6]. Pre-hospital delay consists of the patient-related delay (time from onset of pain to emergency call) and system-related delay (EMS activation delay, travel time to the patients site, and transportation delay to the PCI-capable hospital). Transportation delay deserves attention, as it is a remarkable time-consuming factor in the total pre-hospital delay and the STEMI rescue chain [7, 8].

With about 1.9 million inhabitants, the city of Vienna is the 12th largest city of Europe [9]. We and others have previously described predictors of a prolonged patient-related delay in STEMI, such as age, diabetes, female gender, and low neighborhood household income [10–12]. However, less is known about predictors of transportation delay in acute STEMI patients, as there are few studies that distinguished between transportation delay and overall system-related delay [6, 7]. Moreover, to the best of our knowledge, no comprehensive analysis of the association of the patient’s site (city district) and transportation delay has been performed so far in a large-scale cohort of urban STEMI patients.

Hence, the aim of this study was (1) to test for potential circadian, day-of-week and seasonal variability in transportation times and (2) to investigate the association between transportation delay and patient’s site (defined as district within the city of Vienna, where the emergency call was performed) in our metropolitan Viennese STEMI network.

## Methods

### Patients

The study population consisted of 4751 consecutive patients within a previous period of 8 years who called into the Viennese ambulance service due to ongoing chest pain, in whom STEMI was suspected after the initial onsite ECG, who were immediately transferred to a tertiary hospital for primary PCI, and in whom all necessary variables had been collected. The Vienna-STEMI network was established in 2003 and is composed of the Viennese EMS and six alternating tertiary cardiac centers offering a 24-h primary PCI service for patients with acute STEMI. The EMS is equipped with a 12-lead ECG, devices for advanced life support, and is staffed either with physicians or with paramedics, who will call for an emergency physician if required who arrives at the patient’s site by car in parallel. Usually, the emergency physicians of the Viennese ambulance system (in the majority especially trained general practitioners but also physicians with other expertise) are responsible for diagnosis of STEMI and for choice of reperfusion strategy [13]. The following variables were recorded by co workers of the Viennese EMS: age, gender, time, and date of the emergency call, location of the patient’s site of call (i.e., the district of Vienna), time of arrival of the ambulance at the patient’s site (call-to-site time), time of call until arrival at the hospital (usually the emergency room or the catheter lab of a tertiary department, total transportation time = call-to-door time, a composite of call-to-site, and site-to-door), respectively. Arrival of ambulance at site was defined as the time when the ambulance was stopping at the patient’s door with personnel stuff getting off the ambulance car. Patients above or equal to 75 years were defined as elderly in accordance with the previous studies [14].

### Statistics

Transportation times are presented as minutes [median with interquartile range (IQR)]. Mann–Whitney *U* test was used to test for differences in transportation times between genders and elderly versus younger patients. Kruskal–Wallis test was used to test for between-group difference in transportation times concerning patient’s site, daytime, weekday, and month. Linear regression modelling was performed by entering gender, age, patient’s site, month, weekday, and hour into three models with call-to-site, site-to-door, and call-to-door as the dependent variables, respectively. As logistic regression is a mean-based operation, logarithmic transformation was performed for the variables call-to-site, site-to-door, and call-to-door using the log to base 2 [computed by log2delay = ln(delay)/ln(2)]. The level of significance used for all tests was a *p* value of 0.05. Calculations were performed using SPSS 23, IBM for windows.

## Results

Transportation times were recorded in 4751 patients with suspected STEMI [3093 males (65.1%), mean age 64.5 years]. Baseline characteristics are shown in Table 1. Median call-to-site, site-to-door, and call-to-door times were 9 (7–12), 39 (31–48), and 49 (41–59) min, respectively (Table 1). Women had similar call-to-site times [9 (7–12) min, *p* = 0.114], but significantly longer site-to-door [41 (32–50) min, *p* < 0.001] and call-to-door delays [51 (42–60) min, *p* < 0.001] as compared to men. Elderly patients (≥ 75 years) had slightly, but significantly shorter call-to-site times [9 (7–11) min, *p* = 0.005], but prolonged site-to-door [40 (33–49) min, *p* < 0.001] and call-to-door [50 (42–58) min, *p* = 0.001] delays compared to their younger counterparts.

**Table 1:** Transportation times

	All patients	Females	Males	*p* value	Age < 75	Age >=75	*p* value
Patients’ characteristics [*n* (%)]	4751 (100)	1658 (34.9)	3093 (65.1)	–	3411 (71.8)	1340 (28.2)	–
Call-to-site [minutes, median (IQR)]	9 (7–12)	9 (7–12)	9 (7–12)	0.114	9 (7–12)	9 (7–11)	*p* = 0.005
Site-to-door [minutes, median (IQR)]	39 (31–48)	41 (32–50)	39 (31–48)	< 0.0001	38 (30–47)	40 (33–49)	*p* < 0.001
Call-to-door [minutes, median (IQR)]	49 (41–59)	51 (42–60)	48 (40–58)	< 0.0001	48 (40–58)	50 (42–58)	*p* = 0.001

**Fig. 1:**
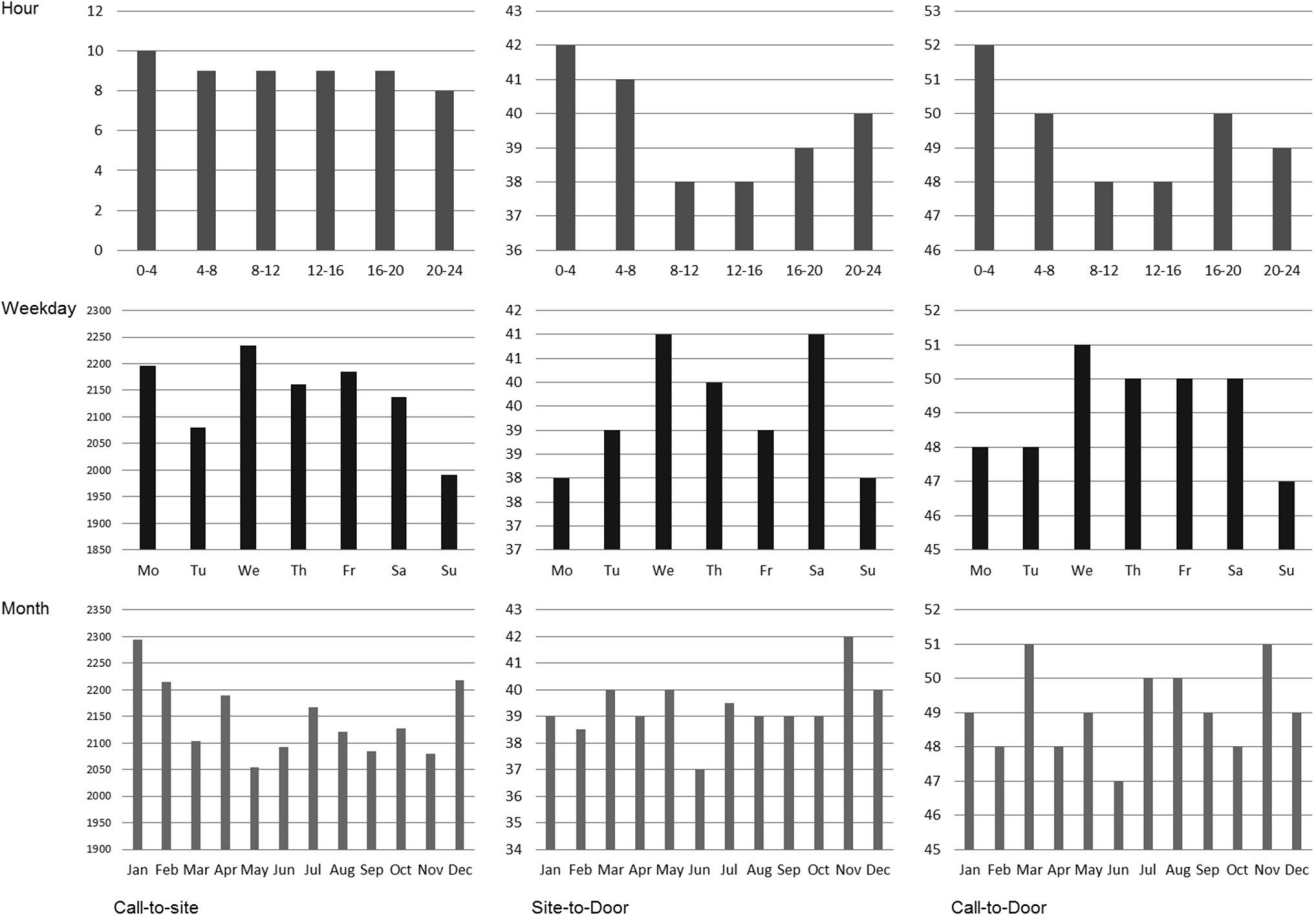
Transportation times according to daytime, weekday, and month. Transportation times (minutes) are shown according to hour of the day (clusters of 4 h, upper row); weekday (middle row); and month (bottom row). Left column: patient call to ambulance arrival at site (call-to-site); mid column: ambulance arrival at site-to-door; right column: patient call-to-door. Call-to-site, site-to-door, and call-to-door were prolonged between 00:00 and 04:00 (*p* < 0.001); the shortest call-to-site, site-to-door, and call-to-door were on Sundays (call-to-site: *p* = 0.013, site-to-door: *p* < 0.001, call-to-door: *p* < 0.001). Month did not significantly impact transportation times (*p* > 0.05) (note: call-to-site for weekday and month is shown as mean rank (as median minutes are equal for each day and month, i.e., 9 min)

The association of daytime, day-of-week, and month on EMS delay is depicted in Fig. 1. Call-to-site [10 (8–12) min], site-to-door [42 (34–51) min], and call-to-door [52 (44–61) min] were significantly prolonged between midnight and 04:00, while the shortest site-to-door [38 (29–48) min] and call-to-door times [48 (40–48) min] were noted between 08:00 and 16:00 (*p* < 0.001). A significant between-group difference in transportation times was found between weekdays, with the shortest call-to-site [9 (7–11) min], site-to-door [38 (31–47) min], and call-to-door [47 (40–57) min] on Sundays, and the longest call-to-site [9 (7–12) min], site-to-door [41 (33–51) min], and call-to-door [51 (43–61) min] on Wednesdays (between-group difference for call-to-site: *p* = 0.013, site-to-door: *p* < 0.001, call-to-door: *p* < 0.001; Fig. 1b). After multivariable adjustment, hour of activation was a strong independent predictor of call-to-site (B − 0.007; 95% CI − 0.01 to − 0.003; *p* = 0.001), site-to-door (B − 0.004; 95% CI − 0.007 to − 0.001; *p* = 0.009), and call-to-door (B − 0.003; 95% CI − 0.005 to − 0.001; *p* = 0.007), while weekday was a determinant of call-to-site only (B − 0.015; 95% CI − 0.027 to − 0.004; *p* = 0.009). Month did not independently influence transportation times (Table 2, Fig. 1c).

**Table 2:** Multivariable predictors of call-to-site, site-to-door, and call-to-door delay

Dependent Variable	Variable	Coefficient (unstandardized)	95% —confidence interval	*p* value
Call-to-site delay	Male gender	0.019	− 0.032 to 0.070	0.459
	Age	− 0.002	− 0.003 to − 0.0002	0.03
	Patient’s site	0.006	0.003 to 0.01	< 0.001
	Weekday	− 0.015	− 0.027 to − 0.004	0.009
	Month	− 0.006	− 0.013 to 0.0002	0.056
	Hour	− 0.007	− 0.01 to − 0.003	0.001
Site-to-door delay	Male gender	− 0.038	− 0.081 to 0.005	0.083
	Age	0.003	0.002 to 0.004	< 0.001
	Patient’s site	0.002	− 0.001 to 0.005	0.191
	Weekday	0.006	− 0.003 to 0.016	0.185
	Month	0.001	− 0.005 to 0.006	0.752
	Hour	− 0.004	− 0.007 to − 0.001	0.009
Call-to-door delay	Male gender	− 0.033	− 0.062 to − 0.003	0.032
	Age	0.002	0.001 to 0.002	0.001
	Patient’s site	0.002	− 0.0001 to 0.004	0.062
	Weekday	− 0.001	− 0.007 to 0.006	0.839
	Month	− 0.002	− 0.006 to 0.002	0.349
	Hour	− 0.003	− 0.005 to − 0.001	0.007

**Fig. 2:**
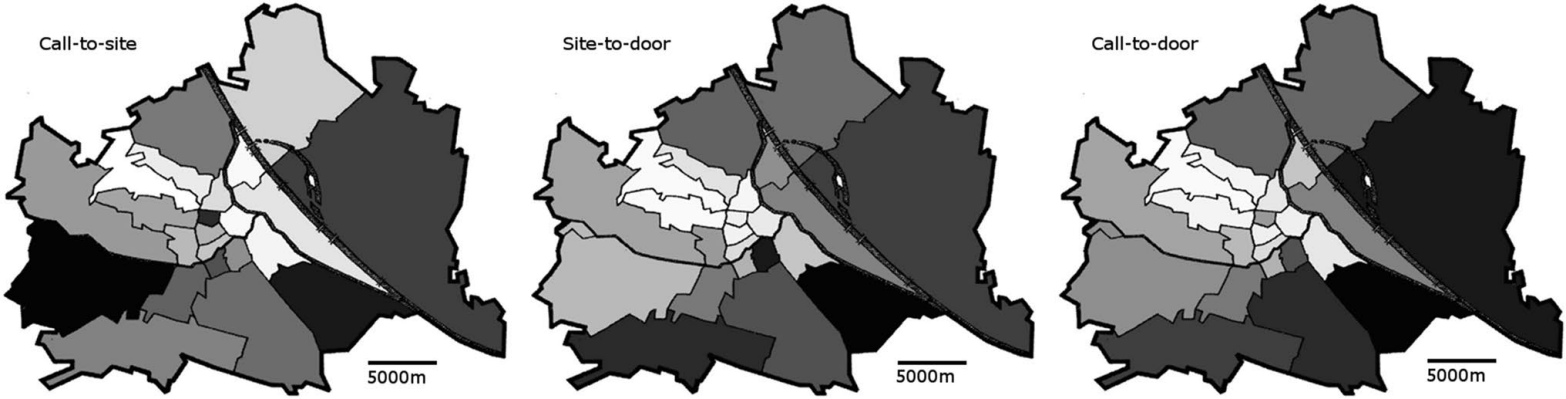
Call-to-site, site-to-door, and call-to-door in the 23 districts of Vienna. Districts are ranked and dyed according to detected transportation times. Dark shade depicts longer delay. Grey dots indicate the location of the six tertiary PCI units

Location of patient’s site (defined as city district) had a significant impact on transportation times, which were shorter in Central and Western districts of Vienna as compared to Southern and Eastern districts (Fig. 2; between-group difference for call-to-site, site-to-door, and call-to-door: *p* < 0.001). Moreover, after multivariable adjustment, patient’s site was the strongest predictor of call-to-site delay (B 0.006; 95% CI 0.003–0.01; *p* < 0.001) and a non-significant determinant of call-to-door delay (B 0.002; 95% CI − 0.0001–0.004; *p* = 0.062, Table 2). Age was a significant independent predictor of call-to-site (B − 0.002; 95% CI − 0.003 to − 0.0002; *p* = 0.03), site-to-door (B 0.003; 95% CI 0.002 to 0.004; *p* < 0.001), and call-to-door time (B 0.002; 95% CI 0.001 to 0.002; *p* = 0.001), while gender did not impact on call-to-site and site-to-door, but was an independent predictor of call-to-door delay (B − 0.033; 95% CI − 0.062 to − 0.003; *p* = 0.032).

## Discussion

In this large-scale observational study on STEMI patients, diagnosed and transferred to a tertiary center by the EMS, we found significant circadian and day-of-week variability of transportation times with the shortest transportation delays between 8:00 and 16:00 (independent of weekday), and on Sundays. Moreover, after multivariable adjustment, hour of activation and age was the strongest predictors of EMS delay. Although median transportation times were guideline conform in all city districts, the location of patient’s site was a key predictor of the transportation delay even after adjustment for age and gender.

The ESC STEMI guidelines recommend timely transport to a PCI-capable hospital with a goal of 90(− 120) min delay between diagnosis of STEMI and reperfusion therapy [1]. In the Vienna-STEMI network, median EMS-related delay from the patient’s site (corresponding to first medical contact, as defined by the guidelines) to admission at the door of a PCI-capable hospital was 39 min. However, we detected notable differences of transportation times concerning daytime, which were significantly prolonged between midnight and 04:00. Potential explanation for this include human resource availabilities (e.g., the number of physicians and paramedics on duty), prolongation of EMS activation time during night, the number of active call centers (with less active centers at night), the distance of EMS centers from the patient’s location, and longer distance to the active primary PCI-center (as only two tertiary centers are open during nights in the Viennese STEMI network, respectively [13].

Several studies found significant circadian variation associated with the incidence and mortality of acute coronary syndromes [15–17] and out-of-hospital cardiac arrest [18]. Accordingly, STEMI incidence peaks in the morning, which has been mainly attributed to diurnal changes in the hemostatic system and platelet aggregability [19]. Increased demand for EMS transports for cardiac indication was reported during such busy traffic times in the morning [20], but did not result in prolonged delay in our study. This suggests that the Viennese EMS was sufficiently staffed and powered during daytime. Due to the fact that we observed a significant time delay in transportation time of only 4 min during night hours, we assume that such delay has no substantial impact on clinical outcome in our network. This assumption is further supported by the previous work of our network, which found that “off-hour admission” at evenings, nights and weekends had no impact on short- and long-term mortality [21]. This can also be expected, as median transportation delays still stayed within the recommended timelines.

As discussed elsewhere, longer pre-hospital transportation delay is usually associated with shorter door-to-balloon times, which can be explained by pre-information of the catheter laboratory about an arriving STEMI case, which allows a faster and better preparation of catheter facilities and catheter personnel [7]. Such explanation might not necessarily apply to the Viennese STEMI network, as the staff on duty is permanently onsite resulting in a very short activation time of the catheter lab.

Apart from circadian variation, we observed a significant difference in transportation times between different districts within the city. It is well known that bigger distance between patient’s site and primary PCI centers directly translates into transportation delay in STEMI, as has been shown in a rural STEMI network [7]. Ideally, a metropolitan STEMI network should offer comparable transportation times to all STEMI patients by a well-balanced distribution of EMS centers and tertiary hospitals. One explanation for the observed prolonged transportation delay in the outlying suburbs in our study might be urban sprawl, typified by low-density construction, poor street connectivity with longer distances, and structural separation of housing from civic and commercial districts [22]. Urban sprawl was associated with delayed EMS response time following motor-vehicle crashes in the United States [23]. In our study, Southern and Eastern districts of the city exhibited prolonged EMS response times, as compared to Central or Western areas as a direct result of bigger distance between patient`s site, and EMS centers and tertiary hospitals, which is shown in Fig. 2. Despite these differences, median transportation delay was within the recommended range in all areas of the Vienna-STEMI network, which guarantees an adequate time to reperfusion for the majority of STEMI patients.

In the present analysis, women had similar call-to-site, but significantly longer site-to-door and call-to-door times compared to men. After multivariable adjustment (for patient’s location, weekday, month, and hour of activation), age and gender remained independent predictors of call-to-door delay. Female patients are typically older, and elderly patients exhibit more comorbidities [24, 25]. Myocardial infarction is complicated by cardiogenic shock more often in these patients [26]. These comorbidities and hemodynamic instability usually go along with prolonged pre-hospital transportation delay [11].

## Limitations

We only included patients in whom primary PCI after direct transportation to a PCI-capable hospital was the reperfusion strategy of choice. Therefore, our results cannot be transformed to STEMI patients who receive pharmacological reperfusion (< 3% in our network) or no reperfusion at all (if contra-indications exist against both reperfusion strategies), or to those who were initially admitted to referral hospitals, which usually prolongs onset of pain-to-door times unacceptably. Moreover, it was our goal to investigate in this trial potential factors that influence the EMS-related transportation part of the pre-hospital delay exclusively. Of note, we were able to adjust for gender and age, but not for other clinical variables or comorbidities that might have influenced our results, as they were not documented by the EMS system on a regular basis.

## Conclusion

In the Vienna-STEMI network, median transportation times are within the international guidelines independent of weekday and daytime. Within these guideline-conform delay times, hour of activation, patient’s site, and age were the strongest determinants of small, but significant transportation delays following an emergency call. The data show that (1) it is unlikely that the existent median transportation delay times have an impact on clinical outcome in the Viennese STEMI system of care and (2) further improvements of the quality of this network might rather concern the reduction of the patient-related delay (until call into the system) and/or a further improvement of in-hospital organization, e.g., a direct transfer of presumable STEMI patients into the catheter laboratory by bypassing emergency rooms or intensive care units. This analysis also demonstrates the importance of performing a registry to optimize patient care where possible. These data from the Viennese STEMI network might also stimulate control of out-of-hospital STEMI management in other cities and networks.
